# Interest and limits of assessing acute kidney injury in administrative dataset

**DOI:** 10.1186/s13613-019-0535-6

**Published:** 2019-05-27

**Authors:** Michaël Darmon, Emmanuel Canet, Lara Zafrani

**Affiliations:** 1 Medical ICU, AP-HP, Saint-Louis University Hospital, 1 Avenue Claude Vellefaux, 75010 Paris, France; 2 Paris-Diderot Medical School, University of Paris, Paris, France; 3 ECSTRA Team, Biostatistics and Clinical Epidemiology, UMR 1153 (Center of Epidemiology and Biostatistic, Sorbonne Paris Cité, CRESS), INSERM, Paris, France; 4 Medical ICU, Hôtel-Dieu University Hospital, Nantes, France; 5 INSERM UMR-976, Paris, France

We read with great interest the manuscript published by Hwang et al. [1]. This later reports a large national cohort of patients from 2008 to 2015 and suggests an independent increase in rate of acute kidney injury (AKI) through the study period and a trend toward a lower mortality [1]. These data are important, and a limited number of manuscripts assessed both change in incidence and prognosis of AKI over time [2]. However, significant limitations deserve to be noted and taken into account. First, these data arise from a national administrative dataset, which has displayed limited reliability. Hence, in line with findings in most of administrative dataset, major diagnosis coding reliability was found to be around 70%, limiting confidence that may be put in both variables of interest and confounding variables adjusted for [3]. Along this line, AKI coding has proven being particularly insensitive, with sensitivity ranging from less than 10 to 60% [4]. Interestingly, AKI code reliability was found to be strongly associated with AKI severity, hospital unit and even gender. For example, codes for renal replacement therapy (RRT)-requiring AKI have higher sensitivity than codes for non-RRT-requiring AKI [4]. As most AKI patients do not require RRT, coding is highly dependent on AKI recognition, coding practices and case-mix [4]. In this line, change in coding reliability over time and change in coding practices induced by greater awareness of AKI conditions, along with changes in practices in terms of renal replacement therapy, are likely to further influence data clustering and to account for part of the observed changes in incidence and outcome [5]. Hence, increasing rate of mild AKI ultimately coded as consequences of increased awareness is likely to lead to a decreased mortality induced by change in overall AKI severity in the later years.

Nonetheless, although coding-based identification of AKI is imperfect and lacks sensitivity, its specificity is fairly robust, and use of administrative dataset is a pragmatic approach to appreciate changes at national level. The study of Jang et al., although hypothesis generating, is important and further underlines need for high quality, worldwide, assessment of AKI epidemiology and of its changes over time. Ideally, these data should be adjusted for clustering effect induced by changes in practices, changes in AKI definition and heterogeneity across centers and countries, along with usual confounders. Accurate assessment of population incidence of AKI is needed to appreciate the public health burden of AKI. More research is needed to address reasons for underlying disparities among groups and reasons behind the increase in the incidence of AKI. It will also help us to understand whether AKI outcome has improved over the last decades, in way to settle achievable goals for the forthcoming years.

## Data Availability

Not applicable.
